# Effect of flavonoids identified in grape pomace extract on efflux pump in *Staphylococcus aureus*

**DOI:** 10.3389/fmicb.2026.1778400

**Published:** 2026-06-19

**Authors:** Loreto Sanhueza, Pablo A. Ortiz, Carolina Arriaza-Echanes, Gabriel I. Krüger, Ricardo Melo, Milena Cotoras, Leonora Mendoza

**Affiliations:** 1 Núcleo de Química y Bioquímica, Facultad de Ciencias, Ingeniería y Tecnología, Universidad Mayor, Santiago, RM, Chile; 2 Departamento de Química, Facultad de Ciencias, Universidad de Chile, Santiago, Chile; 3 Centro de Nanotecnología Aplicada, Facultad de Ciencias, Ingeniería y Tecnología, Universidad Mayor, Huechuraba, Chile; 4 Facultad de Ingeniería y Negocios, Universidad de las Américas, Providencia, Chile; 5 Laboratorio de Micología, Facultad de Química y Biología, Universidad de Santiago de Chile, Santiago, Chile

**Keywords:** efflux pump inhibitors, efflux pumps, flavonoids, grape pomace, *Staphylococcus aureus*

## Abstract

Overexpression of efflux pumps, is a primary mechanism of multidrug resistance in *Staphylococcus aureus*. This study evaluated grape pomace extracts and their constituent flavonoids as potential efflux pump inhibitors (EPIs) to restore ciprofloxacin susceptibility. RP-HPLC analysis of the extracts identified quercetin, (+)-catechin, and (−)-epicatechin. The synergistic interaction between these agents and ciprofloxacin was assessed using the checkerboard method against three *S. aureus* strains: NCTC 8325–4 (*norA* wild type), K2378 (overexpressing *norA*), and K1902 (*norA* deletion). Additionally, ethidium bromide accumulation assays were conducted to quantify pump inhibition activity. All extracts and identified flavonoids exhibited synergism with ciprofloxacin, yielding Fractional Inhibitory Concentration Indices (FICI) ranging from 0.076 to 0.281. While crude extracts demonstrated nonspecific pump inhibition, the isolated flavonoid (−)-epicatechin showed inhibitory activity against the NorA effux pump. Conversely, (+)-catechin and quercetin displayed lower, concentration-dependent activity. These findings demonstrate that grape pomace flavonoids, particularly (−)-epicatechin, act as effective EPIs capable of efflux pumps mediated resistance, suggesting their potential utility as adjuvants in antimicrobial therapy.

## Graphical abstract

**Figure:**
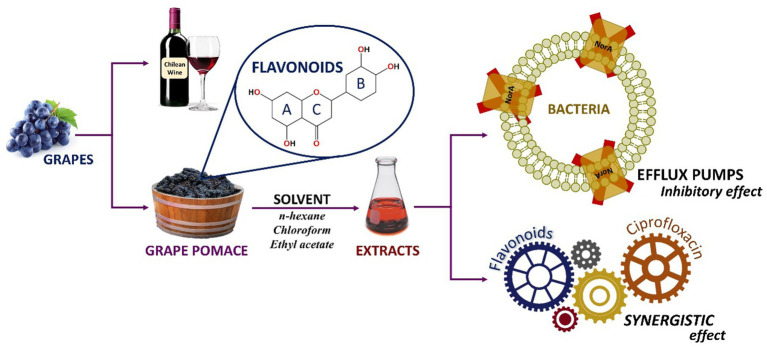

## 1 Introduction

Antimicrobial resistance (AMR) has emerged as one of the most pressing global public health threats of the 21st century (Ahmed et al., 2024). Currently, there are more than 15 classes of antibiotics whose sites of action are related to physiological or metabolic functions essential for bacteria. Unfortunately, none of them has escaped the phenomenon of resistance (Bo et al., 2024). An increasing number of pathogenic bacteria are showing a multi-antibiotic resistance (MDR) phenotype (Ahmed et al., 2024; Bo et al., 2024). This phenotype can be caused by the simultaneous presence of different resistance mechanisms, either in mobile elements or in the bacterial chromosome (Ho et al., 2025). The MDR resistance was primarily attributed to the overuse or unsuitable use of these drugs in various contexts, mainly in clinical treatment, agricultural practices, animal health, and the food system (Bo et al., 2024). Globally, direct deaths related to antibiotic resistance are estimated to have exceeded 1.2 million in 2019, and approximately 10 million deaths are predicted annually by 2050 if adequate measures are not implemented to curb this growing problem (Ho et al., 2025). *Staphylococcus aureus* stands out as the most dangerous superbug among Gram positive microorganisms (Foster, 2004). *S. aureus* is a widely spread opportunistic pathogen that can cause various diseases, including skin infections, endocarditis, osteomyelitis, and sepsis, with outcomes ranging from mild to life-threatening (Brdová et al., 2024). Due to its close association with hospital-acquired infections, *S. aureus* has always been among the first bacterial species to develop resistance to multiple antibacterial agents (Thurlow et al., 2012). An example of this is methicillin-resistant *S. aureus* (MRSA), recognized as an important cause of nosocomial infections and one of the main MDR Gram-positive pathogens with a high mortality rate (Brdová et al., 2024). Among the main resistance mechanisms that bacteria must evade the toxic effects of antibiotics are: (i) inactivation of the antibiotic, (ii) modification of the target site or objective, (iii) decrease in the permeability of the cytoplasmic membrane, (iv) formation of biofilms, and (v) overexpression of efflux pumps (Belay et al., 2024).

Efflux pumps are considered the first line of defence of bacteria against antibiotics. These systems are made up of transport proteins that are integrated into the cytoplasmic membrane and that promote the elimination of toxic compounds from the cytoplasm to the extracellular medium, without the alteration or decomposition of the compound (Belay et al., 2024; Dini, 2026). In general, pumps capable of ejecting multiple structurally unrelated drugs, including antibiotics of different classes, are associated with the MDR phenomenon. Examples of this type of pump in *S. aureus* are NorA, MepA, QacA, MsrA.

The substrates for each specific pump are different and depend on the bacterial species. In general, in bacteria that overexpress MDR efflux pumps, the substrate types include antibiotics such as chloramphenicol, quinolones (nalidixic acid), fluoroquinolones (ciprofloxacin, norfloxacin) and tetracyclines. In addition to acriflavine, ethidium bromide, sodium dodecyl sulphate (SDS), Triton X-100, cetrimide and triclosan (Piddock, 2006).

NorA is the most studied pump in *S. aureus*, the *norA* gene encodes a 42 kDa protein of the bacterial cell membrane, it is chromosomally encoded and expressed at the basal level (Zimmermann et al., 2019). NorA is involved in *S. aureus* resistance against multiple structurally unrelated compounds. NorA substrates include hydrophilic fluoroquinolones such as norfloxacin and ciprofloxacin, dyes (ethidium bromide, rhodamine, acridine, and acriflavine), biocides, and quaternary ammonium salts (Ferreira et al., 2019; Felicetti et al., 2018).

Efflux pump inhibitors are therapeutic agents intended to restore the activity of commonly used antibiotics in clinical practice (Piddock, 2006). Efflux pump inhibitors (EPIs) are molecules that inhibit efflux pumps through one or more mechanisms, leading to the inactivation of drug transport and potentially resulting in the effective accumulation of an antibiotic within a cell (Sharma et al., 2019).

Several chemically synthesized efflux pump inhibitors (EPIs) have been described in the literature, including carbonyl cyanide-*m*-chlorophenyl hydrazone (CCCP), dinitrophenol (DNP) (Zechini and Versace, 2009; Gaurav et al., 2023), and reserpine, among others. However, these compounds exhibit cytotoxic or neurotoxic effects, or their effective concentrations exceed recommended doses. Therefore, due to these problems, EPIs derived from natural sources have been studied (Piddock, 2006; Zechini and Versace, 2009; Gaurav et al., 2023).

Reserpine, an alkaloid isolated from the roots of *Rauwolfia vomitoria*, showed inhibitory activity against the BmR pump in *B. subtilis* (Gibbons, 2024; Dashtbani-Roozbehani and Brown, 2021). Caffeoylquinic acid isolated from *Artemisia absinthium* inhibits transporters belonging to the MFS family (Fiamegos et al., 2011), and diterpenes isolated from *Licopus europaeus* inhibits Tet(K) and Mrs.(A) in *S. aureus* (Gibbons, 2024; Seukep et al., 2020), in addition to totarol isolated from many conifer species inhibits different efflux pumps in *S. aureus* (Seukep et al., 2020; Smith et al., 2007). Furthermore, phenolic compounds have been described as able to inhibit the NorA efflux pump in *S. aureus*. Examples of these compounds are: baicalein isolated from *Scutellaria baicalensis* (Chan et al., 2011; Zhang et al., 2023), biochanin A, chrysoplethin isolated from *Artemisia annua* (Tegos et al., 2011), 5′-methoxy-hydnocarpine (5’-MHC), a flavonoid isolated from *Berberis fremontii* (Dashtbani-Roozbehani and Brown, 2021; Stermitz et al., 2000) as well as kaempferol (Gibbons, 2024; Zhang et al., 2023). While epigallocatechin gallate (EGCg) was able to increase tetracycline concentration in *S. aureus* strains by inhibition of TetK and MsrA (Gibbons, 2024; Seukep et al., 2020; Zhang et al., 2023).

A rich source of phenolic and polyphenolic compounds with antibacterial activity is the grape (*Vitis vinifera*) (Xia et al., 2010; Manca et al., 2020). The main organic waste generated in the wine industry is grape pomace (Rockenbach et al., 2011; Lopes et al., 2025), which represents between 13 and 20% of the total weight of the processed grapes (do Prado Silva et al., 2023). It is produced after the grape crushing, pressing, and fermentation process used to make red wine. Its main components are crushed skin, pulp residue, stems, and seeds (Lopes et al., 2025; Oliveira et al., 2013). Grape pomace is a very important source of various phenolic compounds, due to the poor extraction of these compounds during winemaking (Manca et al., 2020), which mainly include: phenolic acids (gallic, cinnamic, protocatechuic, etc.), flavan-3-ol derivatives, the main ones being: proanthocyanidins (procyanidin dimers B1-B5, procyanidin C1, (+)-catechins, (−)-epicatechin, (−)-epicatechin-3-O-gallate) and flavonoids (Tegos et al., 2011; Stermitz et al., 2000; Mendoza et al., 2013; Almanza-Oliveros et al., 2024). The content of this type of compound in the grape depends on different factors, such as the variety of the wine, environmental factors, harvest, and extraction conditions (do Prado Silva et al., 2023).

Given that phenolic EPIs have been described and the high presence of flavonoids in grape pomace, the objective of this work was to evaluate grape pomace extracts and the flavonoids identified in the pomace as potential EPIs against efflux pumps in *S. aureus,* such as NorA.

## 2 Materials and methods

### 2.1 Grape pomace extracts

To obtain the extracts, a mixture of grape pomace belonging to the Cabernet Sauvignon, Merlot, and Carmenere grape varieties was used. The samples were obtained from the Miguel Torres Vineyard (Curicó, Chile). The pomace samples (900 g) were ground and extracted as described by Mendoza et al. (2013). Briefly, previously ground grape pomace samples were extracted with ethanol 70% (v/v) for 12 h at 4 °C with constant stirring. The extracts obtained were concentrated in a rotary evaporator (BÜCHI model 461) and then subjected to sequential liquid–liquid extractions with the following solvents: *n*-hexane (Merck), chloroform (Merck), and ethyl acetate (Merck). Finally, the fractions obtained (*n*-hexane, chloroform, and ethyl acetate) from each pomace sample were concentrated to dryness and kept at −20 °C.

### 2.2 Reversed-phase high-performance liquid chromatography (RP-HPLC) analysis of grape pomace extracts

a) Chromatographic conditions: the compounds from the grape pomace extracts obtained with *n*-hexane, chloroform, and ethyl acetate were identified by RP-HPLC as described by Mendoza et al. (2013). A Waters 600 HPLC chromatograph (Waters, Mildford, MA, USA) equipped with a diode array detector (Waters 2,990) and a C-18 column (3.9 mm x 150 mm; Waters, Mildford, MA, USA) was used. Chromatography was carried out at 25 °C. The mobile phase and gradient program are shown in Table 1. The run time for each sample was 60 min, the flow rate was 0.8 mL/min, and all measurements were performed at 280 and 360 nm and in triplicate.
b) Sample preparation and compound identification: stock solutions of each extract were prepared at a concentration of 5 mg/mL, and the samples were then dissolved in 1 mL of methanol. The equipment was injected with 20 μL of each extract. The flavonoids present in the extract were identified by comparing the retention times and UV–Vis spectra of the standards kaempferol (≥97.0% degree of purity), quercetin (≥95% degree of purity), (+) catechin (≥98% degree of purity) and (−) epicatechin (≥98% degree of purity). All standards were obtained from Sigma-Aldrich.

**Table 1:** Gradient program.

Time (min)	Acetic acid (1%)	Acetonitrile (100%)
0	90	10
20	80	20
40	80	20
45	50	50
60	50	50

### 2.3 Bacterial strains and culture conditions

The strains used in this study were the *S. aureus* NCTC-8325-4 (*norA* wild type), parental strain for K1902 and K2378. *S. aureus* K1902 (NCTC 8325–4 *norA* deletion) and *S. aureus* K2378 (*norA* overexpressed). The bacterial strains were obtained from the Laboratorio de Microbiología Básica y Aplicada, Universidad de Santiago de Chile collection. Bacterial cultures were grown in Mueller Hinton (MH) broth (Sigma Aldrich, U.S.) and Luria-Bertani (LB) (Sigma-Aldrich, U.S.) media at 37 °C with agitation (220 rpm). *S. aureus* K1902 and *S. aureus* K2378 cultures were supplemented with 10 μg/mL chloramphenicol.

### 2.4 Minimum inhibitory concentration (MIC) determination

The minimal inhibitory concentration (MIC) was defined as the lowest concentration of a compound for which no growth was observed. The MIC determination assays were performed as established by the Clinical and Laboratory Standards Institute (Arriaza-Echanes et al., 2025) guidelines. The two-fold standard micro broth dilution method was performed in 96-well plates. Briefly, bacterial strains were incubated overnight in 3 mL of MH broth at 37 °C with shaking at 220 rpm. The bacterial cultures were then diluted in phosphate-buffered solution (PBS) to McFarland 0.5 (1 × 10^8^ CFU/mL). In each microplate well, 188 μL of MH broth, 10 μL of the extract or flavonoids (140 to 2000 μg/mL), and 2 μL of a bacterial suspension to McFarland 0.5 were added to achieve a final volume of 200 μL. In addition, some wells were used as solvent (dimethyl sulfoxide (DMSO) solvent control) and sterility controls. Microplates were incubated at 37 °C for 24 h, and the optical density was measured at 600 nm (OD_600_) in an Elisa lector (Thermo Scientific Multiskan FC Model, MA, USA). In addition, the MICs of ethidium bromide (EtBr), a universal substrate for efflux pumps, and carbonyl cyanide m-chlorophenyl hydrazone (CCCP), a compound used as a control inhibitor in ethidium bromide accumulation assays, were determined. The MICs of these compounds were also determined using the described method. All results were expressed as μg/mL, and experiments were performed in triplicate in three independent experiments. The results were expressed as percentage inhibition for each of the grape pomace extracts (obtained with *n*-hexane, chloroform, and ethyl acetate). The following controls were used for the MIC tests: the absorbance control: corresponding extract or compounds analyzed was subtracted from each sample absorbance value, solvent control: the bacteria that grew in DMSO (solvent control) were considered 100% in all cases, except ethidium bromide in which the solvent was distilled water.

### 2.5 Checkerboard assays of grape pomace extracts and flavonoids identified

The synergy assays between extract obtained (*n*-hexane, chloroform, or ethyl acetate), or pure flavonoids (quercetin, (+)-catechin, (−) epicatechin) in combination with ciprofloxacin against *S. aureus* strains: NCTC 8325–4 (*norA* wild type), K2378 (overexpressing *norA*), and K1902 (*norA* deletion) were evaluated by the checkerboard method described by Motyl et al. (2006). Briefly, seven serial, twofold dilutions of extract or flavonoid, and ciprofloxacin were prepared. In a 96-well plate, 25 μL of each dilution of extract or flavonoid was added in each vertical row, and 25 μL of the ciprofloxacin dilution was added in each horizontal row. Both the first horizontal and vertical rows were left with only one agent, and the following rows contained a fixed amount of one agent and increasing concentrations of the second agent. In the selection of the range of concentrations, the MICs obtained for each tested agent and tested *S. aureus* strains studied were considered. The extracts and flavonoids concentrations used ranged from 2000 to 2.2 μg/mL, 0.003 to 2 μg/mL for ciprofloxacin. To each well, 100 μL of MH broth and 10 μL bacterial suspension to McFarland 0.5 (1 × 10^8^ CFU/mL) were added. The following controls were used for the checkerboard assays: the absorbance control in which the absorbance of the extract or flavonoid in combination with ciprofloxacin was subtracted from the absorbance value of each sample, growth control (CC), bacteria with only culture medium, solvent control (CS), and a sterility control (CE), which contains only culture medium, were included. Subsequently, the plates were incubated for 18 h at 37 °C. The plates were incubated at 37 °C for 24 h and measured at 600 nm in Elisa lector (Thermos Labsystems Multiskan FC Model). All tests were performed in triplicate in three different experiments.

Fractional inhibitory concentrations (FIC) were calculated the formula FIC_extract or flavonoid_ = (MIC extract or flavonoid + antibiotic / MIC extract or flavonoid) or FIC_antibiotic_ = (MIC extract or flavonoid + antibiotic / MIC antibiotic). The FIC index (FICI) for each combination was calculated by the sum of both FIC values, and results were interpreted as follows: FICI ≤ 0.5 synergic effect, 0.5 < FICI ≤ 4 additive effect, and FICI > 4 antagonistic effects (Motyl et al., 2006). The same formula was used for the calculation of the combinations of extracts or pure flavonoids (quercetin, (+)-catechin, (−)-epicatechin) with the antibiotic studied (ciprofloxacin).

### 2.6 Efflux pump inhibition assays in *S. aureus* strains

To determine whether grape pomace extracts and the identified flavonoids have the capacity to inhibit efflux pumps in *S. aureus*, the activity of the efflux pumps was evaluated through a fluorimetric method described by Viveiros et al. (2008), which uses EtBr as a fluorescent substrate. EtBr has been widely used as a probe for these analyses, since it is able to emit a weak fluorescence intensity in aqueous solutions (outside the cell), while in hydrophobic environments (inside the cell) the fluorescence intensity becomes greater especially as EtBr penetrates the bacterial cell wall and accumulates in the cytoplasm adjacent to the cytoplasmic membrane in Gram-positive bacteria.

a) Preparation of bacterial cultures: for EtBr accumulation assays, the studied *S. aureus* strains: NCTC 8325–4 (*norA* wild type), K2378 (overexpressing *norA*), and K1902 (*norA* deletion). were grown in 10 mL of Luria Bertani (LB) broth until they reached a logarithmic phase corresponding to an optical density at 600 nm (OD_600_) of 0.6. Bacterial cultures were centrifuged at 12,000 rpm (relative centrifugal force (RCF) 6,000) for 3 min, the pellet was collected, and the supernatant was discarded, then the pellet was washed with phosphate-buffered saline (PBS), and the OD_600_ was adjusted to 0.3 in the same buffer. Subsequently, glucose was added to the cell suspension at a final concentration of 0.4% (v/v), and 95 μL aliquots of the suspension were transferred to a 96-well plate.
b) Ethidium bromide accumulation assay: The effects of extracts (*n*-hexane, chloroform, and ethyl acetate) and flavonoids (quercetin, (+)-catechin, and (−)-epicatechin) on the accumulation of EtBr were evaluated in the *S. aureus* strains: NCTC 8325–4 (wild type), K1902 (*ΔnorA*) and K2378 (*norA*++). Sub-inhibitory concentrations (½, ¼, and ⅛ of the MIC) of the extracts or pure compounds were used in all experiments, and CCCP was used as an inhibitor control. The assay was carried out according to Paixão et al. (2009). Briefly, the *S. aureus* strains were grown in 10 mL of LB medium until they reached a mid-log phase, which corresponded to an OD_600_ of 0.6. Then, the bacterial culture was centrifuged at 10,786 × g for 3 min, the pellet was washed twice with the same volume of PBS, and the OD_600_ of the cellular suspension was adjusted to 0.3. Glucose was added to the cellular suspension to achieve a final concentration of 0.6% (v/v), and aliquots of 95 μL were transferred to 0.2 mL wells in 96-well microtiter plates. Aliquots containing 0.4 μL of EtBr or 0.2 μL of CCCP were then added to obtain final concentrations of 1.0 μg/mL and 1.25 μg/mL of EtBr and CCCP, respectively. Fluorescence (530/580 nm excitation/detection) was monitored by a microplate reader (BioTek Synergy HT Model, VT, USA) at 37 °C every 5 min for a period of 1 h. The effects of the extracts and compounds on the accumulation of EtBr were determined under conditions that optimized efflux (i.e., the presence of glucose and incubation at 37 °C). For all tests performed, controls were included in which the bacterial suspension culture medium was replaced with: (1) PBS (2) PBS containing glucose, (3) PBS + EtBr at different concentrations (4) PBS + extract or flavonoid, (5) PBS + extract or flavonoids + EtBr, (6) PBS + CCCP.

### 2.7 Statistical analysis

Statistical analysis for all treatments was performed using two-way ANOVA with a *p*-value < 0.05, using GraphPad Prism 5 software.

## 3 Results and discussion

### 3.1 Chromatographic analysis of flavonoids in grape pomace extracts

To identify the flavonoids in the grape pomace extracts, RP-HPLC was used. Table 2 shows each of the flavonoids identified in the extracts. Only quercetin was identified in the extract obtained with *n*-hexane, while quercetin, (+)-catechin, and (−)-epicatechin were identified in the extracts obtained with chloroform and ethyl acetate.

**Table 2:** Flavonoids identified in grape pomace extracts.

Solvent	Identified compound	Retention time [min]	UV bands [nm]
*n*-hexane	Quercetin	255.5–369.3	52.1
Chloroform	Quercetin	255.5–369.3	52.1
	(+)-catechin	228.4–279.1	13.5
	(−)-epicatechin	227.2–279.1	18.5
Ethyl acetate	Quercetin	255.5–369.3	52.1
	(+)-catechin	228.4–279.1	13.5
	(−)-epicatechin	227.2–279.1	18.5

It is important to note that this work focused on identifying the flavonoids present in grape pomace extracts, as these compounds have been described as possessing efflux pump inhibitor activity. Although other compounds, such as phenolic acids and anthocyanins, have been identified in grape pomace, these were not analysed, since the objective of this work was to search for efflux pump inhibitors (EPIs) from grape pomace.

In fact, previous work by our research group (Mendoza et al., 2013; Sanhueza et al., 2017) and the literature in general extensively describe the presence in grape pomace of phenolic acids such as gallic, vanillic, syringic, and *p*-coumaric acid, among others, as well as flavonoids such as quercetin, kaempferol, (+)-catechin, (−)-epicatechin, and luteolin, among others (Machado et al., 2024; Buican et al., 2025). The content and abundance of these compounds depend on many factors, such as geographical location, climate, and the extraction and identification methods employed (Yammine et al., 2020).

For *S. aureus* efflux pump inhibition assays, sub inhibitory concentrations are required, so the MIC of each of the grape pomace extracts (obtained with *n-*hexane, chloroform and ethyl acetate), of the identified flavonoids (quercetin, (+)-catechin, and (−)-epicatechin), were determined in *S. aureus* strains: NCTC 8325–4 (*norA* wild-type), K2378 (overexpressing *norA*), and K1902 (*norA* deletion). The MICs of each extract or compound are shown in Table 3. The minimum inhibitory concentrations (MICs) of the analysed extracts (*n*-hexane, chloroform, and ethyl acetate) ranged from 500 to 750 μg/mL, while the MICs obtained with the identified pure flavonoids ranged from 140 to 2000 μg/mL in all strains tested. Among the flavonoids, quercetin exhibited the lowest minimum inhibitory concentration, at 140 μg/mL, in all strains studied.

**Table 3:** Analysis of synergism between grape pomace extracts or pure identified flavonoids in the extracts and ciprofloxacin in *S. aureus* strains.

			MIC (μg/mL)			
*S. aureus* strains	Extract or flavonoid	Extract or flavonoid	Ciprofloxacin	Extract or flavonoid plus ciprofloxacin	MIC reduction (fold) of ciprofloxacin	FICI
	*n*-hexane	0500		0.25	8	0.141 (S)
	Chloroform	500		0.25	8	0.156 (S)
NCTC 8325–4 (*norA* wild type)	Ethyl acetate	750		0.25	8	0.156 (S)
	Quercetin	140	2.0	0.13	16	0.188 (S)
	(+)-Catechin	2000		0.25	8	0.078 (S)
	(−)-Epicatechin	2000		0.13	16	0.094 (S)
	*n*-hexane	500		0.21	17	0.076 (S)
	Chloroform	500		0.21	17	0.091 (S)
	Ethyl acetate	750		0.44	8	0.156 (S)
K2378 (*norA*++)	Quercetin	140	3.5	0.44	8	0.159 (S)
	(+)-Catechin	2000		0.44	8	0.141 (S)
	(−)-Epicatechin	2000		0.21	17	0.076 (S)
	*n*-hexane	500		0.063	8	0.142 (S)
	Chloroform	500		0.063	8	0.157 (S)
K1902 (Δ*norA*)	Ethyl acetate	750		0.125	4	0.281 (S)
	Quercetin	140	0.5	0.125	4	0.281 (S)
	(+)-Catechin	1,000		0.125	4	0.281 (S)
	(−)-Epicatechin	1,000		0.125	4	0.281 (S)

FICI indexes were interpreted as: FICI ≤ 0.5 synergic effect, 0.5 < FICI ≤ 4 additive effect, and FICI > 4 antagonistic effect. (S) synergic interaction.

### 3.2 Synergy interactions analysis

For the evaluation of the synergistic effect between grape pomace extracts or pure identified flavonoids in combination with ciprofloxacin, an antibiotic belonging to the fluoroquinolone class and recognized substrate of the NorA efflux pump in *S. aureus*, the checkerboard assay was used (Table 3).

The MIC of ciprofloxacin was lower in the *S. aureus* K1902 (*norA* deletion), which does not have the NorA efflux pump, with a MIC of 0.5 μg/mL, while *S. aureus* NCTC 8325–4 (*norA* wild type) and *S. aureus* K2378 (overexpressing *norA*), has higher MICs for this antibiotic, which were 2.0 and 3.5 μg/mL, respectively.

However, when this antibiotic was combined with each of the extracts (*n-*hexane, chloroform and ethyl acetate) and flavonoids identified, it was observed that all combinations had a synergistic effect on all strains tested with FICI that varied in a range of 0.076–0.281. The observed synergistic effect was reflected in the MIC values. In *S. aureus* NCTC 8325–4 (*norA* wild type strain) the MIC reductions of ciprofloxacin were up to 16-fold when this antibiotic was combined with (−)-epicatechin (FICI = 0.076), while in *S. aureus* K2378 as in *S. aureus* NCTC 8325–4 the greatest reduction in the MIC of ciprofloxacin was observed when it was combined with (−)-epicatechin with a MIC reduction of 17-fold. In *S. aureus* K1902 (*norA* deletion), synergism was also observed in all the combinations tested, being the combination with the greatest effect.

These results agree with reported in the literature in that combinations of plant extracts with antibiotics belonging to different families show synergism against *S. aureus* strains (MSSA, MRSA), considerably reducing the MIC of all the antibiotics tested (Sanhueza et al., 2017; Atta et al., 2023).

Considering the profile of phenolic compounds found in grape pomace and the fact that the phenolic acids and identified flavonoids in the grape pomace extract have distinct mechanisms of action, such as; damage to the cytoplasmic membrane, such as catechins and galangin, capable of disrupting lipid bilayers, since they penetrate and alter the barrier function (Cushniea and Lamb, 2011), in addition to 6-phenylapigenin and 4-hydroxylonchocarpine that depolarize the *S. aureus* membrane (Dzoyem et al., 2013) (2) Nucleic acid synthesis, among the active compounds are: myricetin and (−)-epigallocatechin in *Proteus vulgaris* (Cushniea and Lamb, 2011), quercetin and apigenin (inhibition of DNA gyrase) in *E. coli*, and galangin in *K. pneumoniae* (binding to DnaB helicase) (Chen and Huang, 2011), and by inhibiting some metabolic function essential for the bacteria, such as enzymes that participate in the synthesis of folic acid. It has also been described that (−)-epigallocatechin gallate (EGCg) is able to inhibit the synthesis of penicillin-binding proteins (PBPs) (Wagner and Ulrich-Merzenich, 2009) and in addition, certain flavonoids are able to inhibit bacterial resistance mechanisms, such as: EGCg and baicalein described as beta-lactamase inhibitors in methicillin-susceptible *S. aureus* strains (MSSA) (Liu et al., 2000; Hu et al., 2001; Zhao et al., 2001), and at the same time inhibit TetK, a tetracycline-specific efflux pump in *S. aureus* (Roccaro et al., 2004). Similarly, the flavone 5′-methoxyhydnocarpine is able to inhibit the MDR NorA efflux pump in *S. aureus* (Smith et al., 2007). It is suggested that the synergistic effect of the studied extracts can be explained by a multi-target effect. Therefore, the extract could act at different points, increasing bacterial susceptibility and enhancing antibiotic activity. Another mechanism by which the synergistic effect described is produced is the interaction of certain compounds present in the extracts with bacterial resistance mechanisms, and in this way, they can partially or completely suppress some resistance mechanism, as is the case of the efflux pumps (Wagner, 2011; Hemaiswarya et al., 2008; Vaou et al., 2022). Various flavonoids have been described in the literature as inhibitors of these efflux pumps, thereby reversing resistance to antibiotics that are substrates of these pumps. Examples of these compounds are flavones, flavone derivatives, isoflavones, and flavonoglycans (Handzlik et al., 2013).

### 3.3 Effect of grape pomace extracts on ethidium bromide accumulation on the NorA efflux pump in *S. aureus*

*S. aureus* strain was selected because it is the most important Gram-positive nosocomial pathogen due to its MDR phenotype. *S. aureus* possesses several efflux pumps that contribute to multidrug resistance to antibiotics, including MepA and NorA, the latter of which is widely distributed in this species (Kaatz et al., 2004). For the *S. aureus* efflux pump inhibition assays, sublethal concentrations were needed, so the MIC of BrEt and the control inhibitor (CCCP) were first determined, which are shown in Table 4. The MIC of EtBr was 2 μg/mL in all strains studied. This was because all the strains analysed expressed various types of transport proteins bound to the cytoplasmic membrane in which BrEt is a substrate (Paixão et al., 2009; Zimmermanna et al., 2017). In the case of the *S. aureus norA* wild type strain (parental strain of K1902 and K2378), expresses various efflux pumps for example, MepA and NorA (Kaatz et al., 2004; Kaatz et al., 2000). *S. aureus* K1902 (*norA* deletion), expresed other effux pumps (for example MepA) for which BrEt is a substrate. It is for this reason that the MICs of BrEt in the three strains studied do not varied.

**Table 4:** Minimum inhibitory concentration (MIC) in *S. aureus* strains of the control compounds.

	Minimum inhibitory concentration ((μg/mL)		
Control compounds	NCTC 8325–4 (*norA* wild type)	K2378 (*norA*++)	K1902 (Δ*norA*)
Ethidium bromide	2	2	2
CCCP	2.5	2.5	1.5

### 3.4 Accumulation kinetics of ethidium bromide in *S. aureus* strains

**Figure 1:**
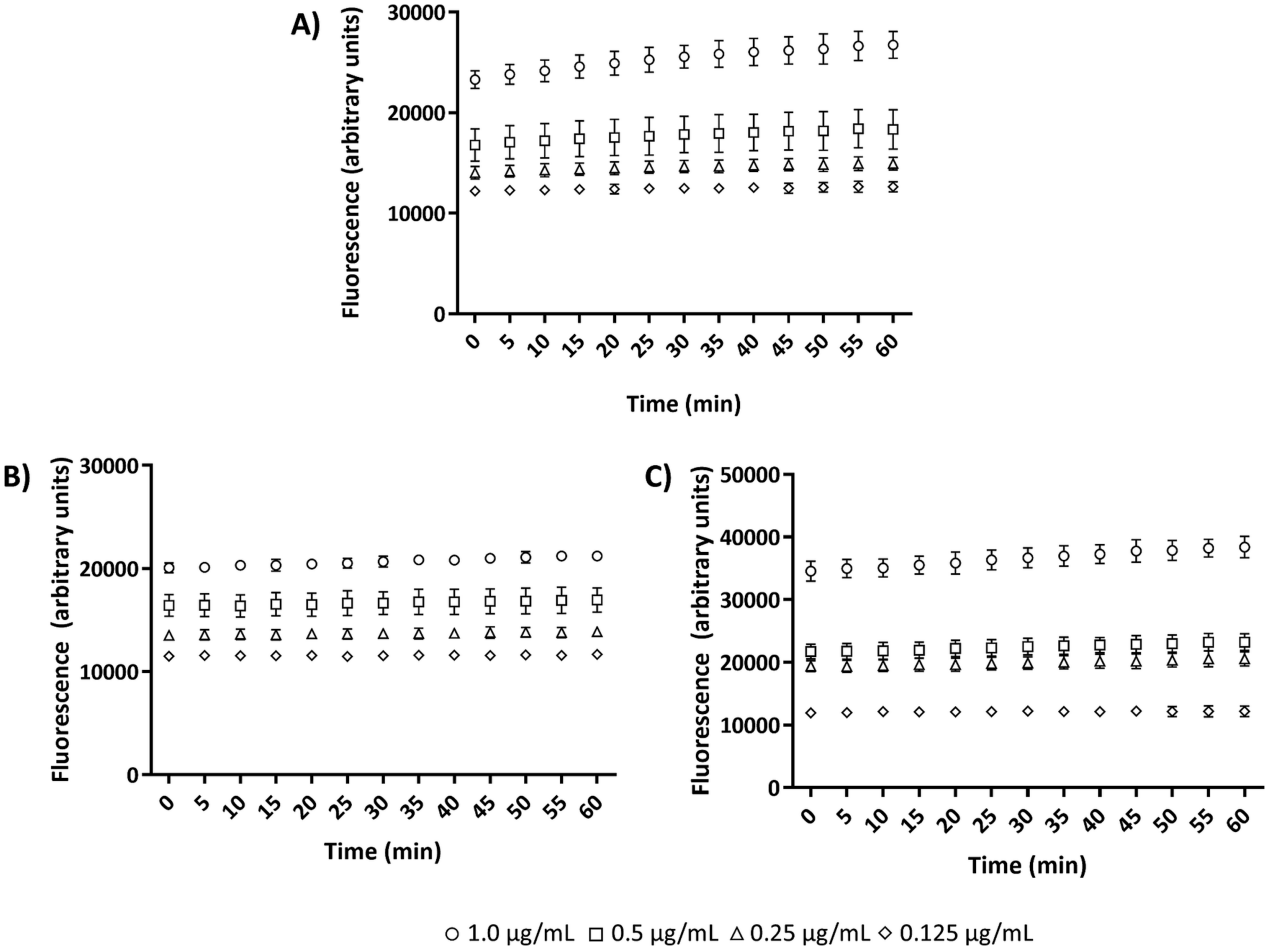
Accumulation kinetics of BrEt in *S. aureus* strains. 1 μg/mL (circles), 0.5 μg/mL (squares), 0.25 μg/mL (triangles), 0.125 μg/mL (rhombuses) in NCTC 8325–4 (*norA* wild type) **(A)**, K2378 (overexpressing *norA*) **(B)**, and K1902 (*norA* deletion) **(C)**. Cells at OD_600_ of 0.6 were washed and incubated with EtBr at 37 °C in the presence of glucose (0.4%), the fluorescence intensity of EtBr was measured at an excitation wavelength of 530 nm, and 600 nm emission, every 5 min for 1 h.

Using the sub-inhibitory concentrations obtained for ethidium bromide (1, 0.5, 0.25, and 0.125 μg/mL), the accumulation of this substrate in *S. aureus* strains: NCTC 8325–4 (*norA* wild type), K2378 (overexpressing *norA*), and K1902 (*norA* deletion) was determined. The accumulation kinetics of BrEt in the three strains are shown in Figure 1, which shows in the figure, the accumulation of BrEt is concentration dependent. As the concentration of BrEt increased, the intensity of the observed fluorescence also increased in all strains tested. These results suggest that the efflux pumps in *S. aureus* strains are active, since this substrate did not accumulate inside the cell, but is being expelled. The strains *S. aureus* NCTC8325-4 (*norA* wild type) and *S. aureus* K2378 (overexpressing *norA*) showed similar readings since, as mentioned above, these strains, in addition to the *norA* efflux pump, possess different active transport proteins in which BrEt is a substrate (Kaatz et al., 2004; Kaatz et al., 2000). In the case of the *S. aureus* K1902 (*norA* deletion), although this strain does not express the NorA efflux pump, it has several active efflux pumps such as MepA and MrsA, which explains the efflux of BrEt (Kaatz et al., 2004).

### 3.5 Ethidium bromide accumulation assays

**Figure 2:**
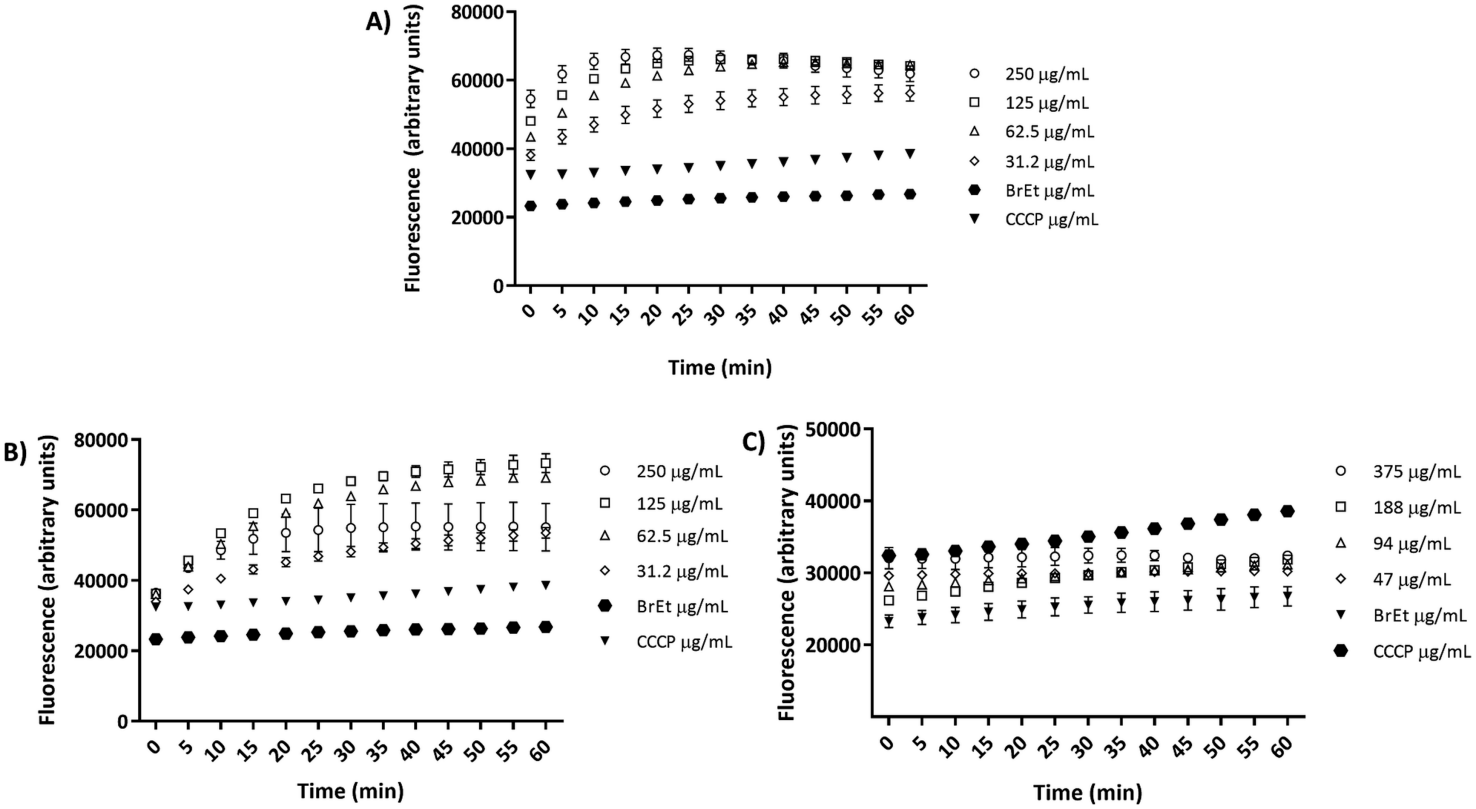
Effect of grape pomace extracts on BrEt accumulation in *S. aureus norA* wild type (NCTC-8325-4). **(A)** Extract obtained with *n*-hexane, **(B)** extract obtained with chloroform, and **(C)** extract obtained with ethyl acetate. The cells at OD_600_ of 0.6 were washed and incubated simultaneously with BrEt (1 μg/mL) and each of the analysed extracts (without exceeding ½ MIC) at 37 °C in the presence of glucose (0.4%), the fluorescence intensity of BrEt was measured at an excitation wavelength of 530 nm and 600 nm emission, every 5 min for 1 h, CCCP (1.25 μg/mL) was used as a control inhibitor.

**Figure 3:**
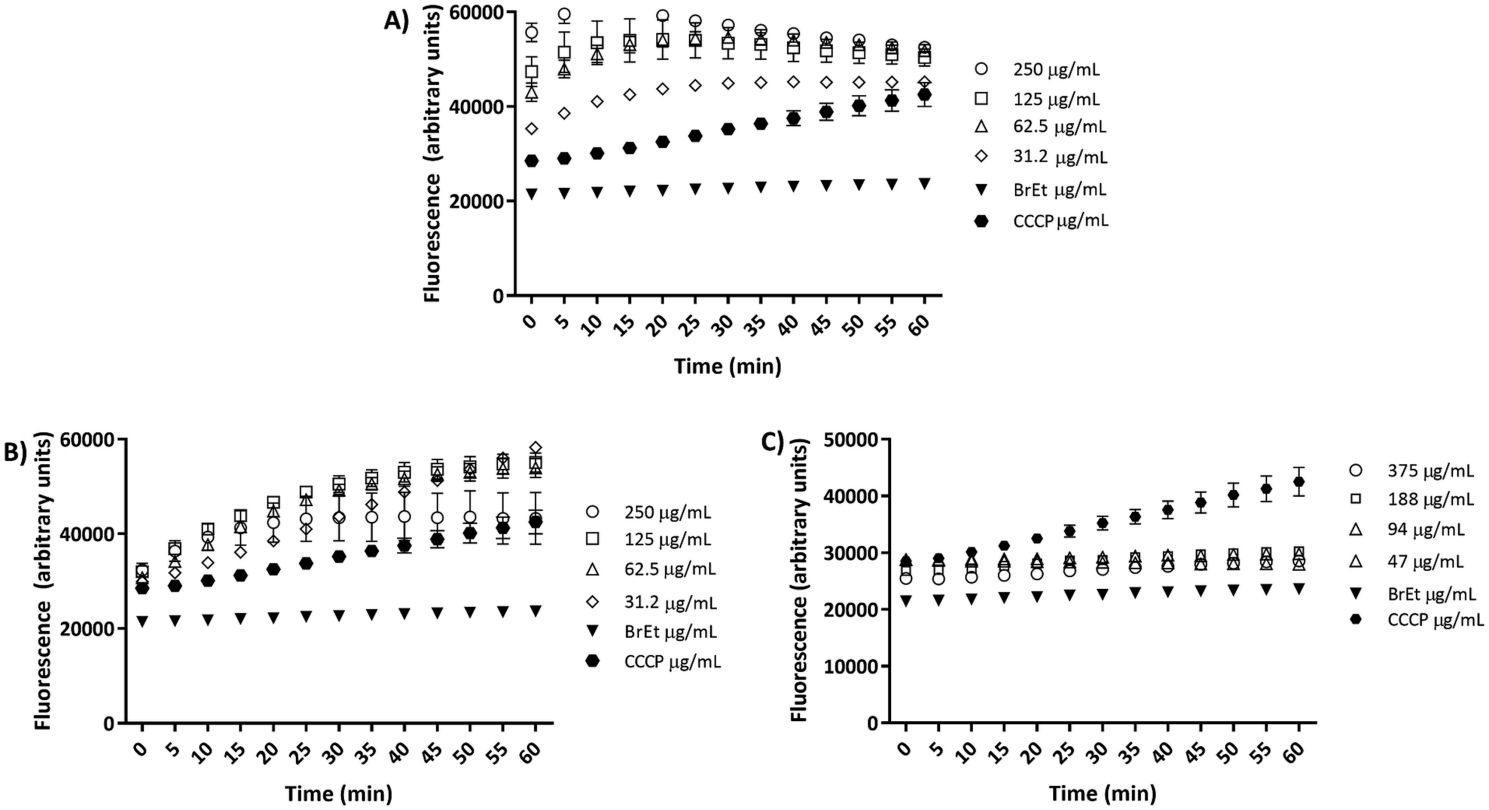
Effect of grape pomace extracts on ethidium bromide accumulation in *S. aureus* overexpressing *norA* (K2378) strain. **(A)** Extract obtained with *n*-hexane, **(B)** extract obtained with chloroform, and **(C)** extract obtained with ethyl acetate. The cells at OD_600_ of 0.6 were washed and incubated simultaneously with BrEt (1 μg/mL) and each of the analysed extracts (without exceeding ½ MIC) at 37 °C in the presence of glucose (0.4%), the fluorescence intensity of BrEt was measured at an excitation wavelength of 530 nm and 600 nm emission, every 5 min for 1 h, CCCP (1.25 μg/mL) was used as a control inhibitor.

**Figure 4:**
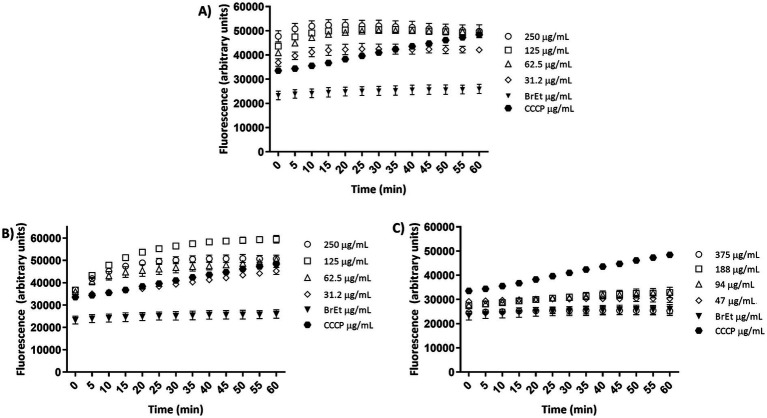
Effect of grape pomace extracts on ethidium bromide accumulation in *S. aureus norA* deletion (K1902). **(A)** Extract obtained with *n*-hexane, **(B)** extract obtained with chloroform, and **(C)** extract obtained with ethyl acetate. The cells at OD_600_ of 0.6 were washed and incubated simultaneously with BrEt (1 μg/mL) and each of the analysed extracts (without exceeding ½ MIC) at 37 °C in the presence of glucose (0.4%), the fluorescence intensity of BrEt was measured at an excitation wavelength of 530 nm and 600 nm emission, every 5 min for 1 h, CCCP (1.25 μg/mL) was used as a control inhibitor.

The effect of different sub-inhibitory concentrations of grape pomace extracts on the NorA efflux pump on EtBr accumulation was evaluated using the *S. aureus* strains: *norA* wild type (NCTC 8325–4), overexpressing *norA* (K2378), and *norA* deletion (K1902). Figures 2–4 show the accumulation of BrEt in bacterial cells increased in the presence of CCCP (EPI control) in all strains studied.

When NCTC 8325–4, K2378, K1902 strains were treated with different sub-inhibitory concentrations of the extracts obtained with *n*-hexane (Figures 2A–4A) and chloroform (Figures 2B–4B), both in a range of 250 to 31.2 μg/mL, the fluorescence intensity increased over time even higher than the control inhibitor (CCCP) at concentrations of 62.5 and 125 μg/mL, respectively.

The *S. aureus* K1902 strain does not possess the gene that codes for the NorA efflux pump; however, it does possess genes that code for other transport proteins such as MepA and MrsA, which, like NorA, use BrEt as one of their substrates, which explains the observed effect (Kaatz et al., 2004). However, when the studied strains were treated with the extract obtained with ethyl acetate, the fluorescence intensity of BrEt was lower than that observed when treating the cells with CCCP ranges that did not exceed (94–375 μg/mL).

These results suggest that within the extract obtained with ethyl acetate, the identified flavonoids may be found in lower concentrations or that the extract contains compounds capable of antagonizing EPI activity. This would explain the lesser effect of this extract on the studied strains compared to the extracts obtained with n-hexane and chloroform. As mentioned previously, the study only considered the identification of flavonoids due to their recognized inhibitory activity on efflux pumps (Liu et al., 2000; Waditzer and Buscar, 2021).

However, among the compounds identified in grape pomace are a wide variety of compounds with diverse chemical structures, such as phenolic acid (Sanhueza et al., 2017), norisoprenoids, terpenes and methoxypyrazines (Buican et al., 2025).

Flavonoids able to inhibit efflux pumps have been described in the literature, such as 5-methoxyhydnocarpine, which is active against the NorA efflux pump present in *S. aureus* (Dashtbani-Roozbehani and Brown, 2021; Stermitz et al., 2000). Furthermore, it has been determined that extracts of *Persea lingue* also have activity against NorA, with kaempferol being the active compound at a concentration of 4.5 μg/mL (Dashtbani-Roozbehani and Brown, 2021).

**Figure 5:**
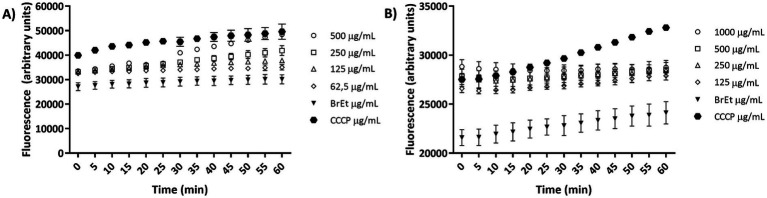
Effect of (+)-catechin on the accumulation of EtBr in *S. aureus* K2378 (overexpressing *norA*) **(A)** and K1902 (*norA* deletion) **(B)** strains. The cells at OD_600_ of 0.6 were washed and incubated simultaneously with BrEt (1 μg/mL) and (+)-catechin (without exceeding ½ MIC) at 37 °C in the presence of glucose (0.4%), the fluorescence intensity of BrEt was measured at an excitation wavelength of 530 nm and 600 nm emission, every 5 min for 1 h, CCCP (1.25 μg/mL) was used as a control inhibitor.

**Figure 6:**
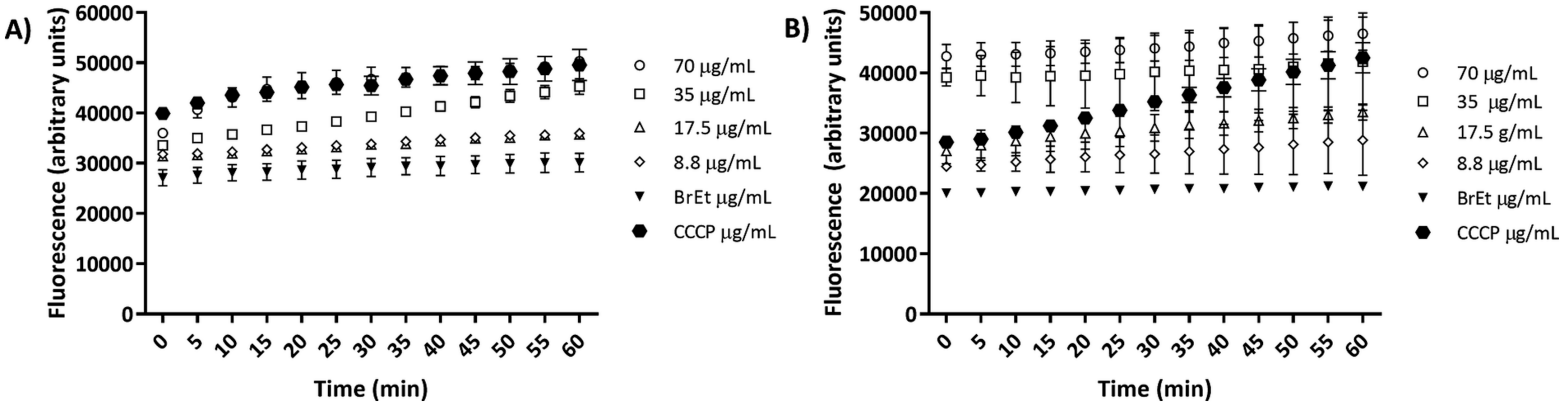
Effect of quercetin on the accumulation of EtBr in *S. aureus* K2378 (overexpressing *norA*) **(A)** and K1902 (*norA* deletion) **(B)** strains. The cells at OD_600_ of 0.6 were washed and incubated simultaneously with BrEt (1 μg/mL) and quercetin (without exceeding ½ MIC) at 37 °C in the presence of glucose (0.4%), the fluorescence intensity of BrEt was measured at an excitation wavelength of 530 nm and 600 nm emission, every 5 min for 1 h, CCCP (1.25 μg/mL) was used as a control inhibitor.

**Figure 7:**
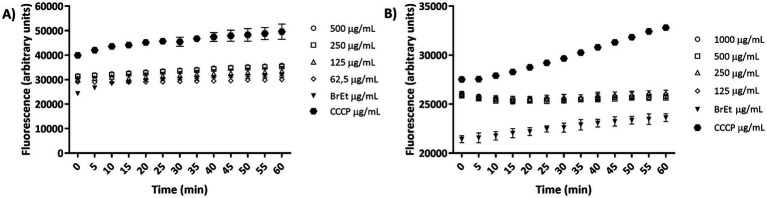
Effect of (−)-epicatechin on the accumulation of EtBr in *S. aureus* K2378 (overexpressing *norA*) **(A)** and K1902 (*norA* deletion) **(B)** strains. The cells at OD_600_ of 0.6 were washed and incubated simultaneously with BrEt (1 μg/mL) and (−)-epicatechin (without exceeding ½ MIC) at 37 °C in the presence of glucose (0.4%), the fluorescence intensity of BrEt was measured at an excitation wavelength of 530 nm and 600 nm emission, every 5 min for 1 h, CCCP (1.25 μg/mL) was used as a control inhibitor.

After the evaluation of the extracts, ability of the identified flavonoids in the grape pomace extracts to inhibit the NorA efflux pump in *S. aureus* was determined. For this, *S. aureus* strains: NCTC-8325-4, K2378 and K1902 were used. The results with (+)-catechin, quercetin, and (−)-epicatechin are shown in Figures 5–7.

The results show that when *S. aureus* K2378 (overexpressing *norA*) was treated with (+)-catechin and quercetin (Figures 5A, 6A), the fluorescence increased over time, indicating that these compounds are potential EPIs. In the case of (+)- catechin, this effect was observed at all concentrations tested (1,000, 500, 250, and 125 μg/mL); however, this effect was not higher than the control inhibitor. When the cells were treated with quercetin, an increase in the fluorescence intensity of EtBr was observed at the highest concentrations (70 and 35 μg/mL), higher than the EPI control, so its inhibitory effect is concentration dependent. In the case of the *S. aureus* K1902 (*norA* deletion) strain, when the cells were treated with (+)-catechin (Figure 5B) the same effect was observed as in the *S. aureus* K2378 (overexpressing *norA*) (Figure 5A) strain, while when they were exposed to quercetin the effect on the accumulation of BrEt was lower, that is, the increase in fluorescence intensity was only comparable to CCCP at the highest concentration tested (70 μg/mL).

The result was different when cells of the *S. aureus* K2378 and *S. aureus* K1902 strains were treated with (−)-epicatechin (Figures 7A,B, respectively). Since this flavonoid showed a specific effect on the *S. aureus* K2378 (Figure 7A) strain, showing an increased in fluorescence intensity not exceeding CCCP but constant at all concentrations tested (1000–125 μg/mL).

This effect was not observed in *S. aureus* K1902 cells (Figure 7B). Since the fluorescence intensity observed at all concentrations (500–62.5 μg/mL) was only similar to the fluorescence intensity when these cells were treated only with BrEt (substrate of the NorA efflux pump). Therefore, it is suggested that (−)-epicatechin is a inhibitor of the NorA efflux pump in *S. aureus*, with an effect similar that observed with the control inhibitor. These results are consistent with those reported in the literature.

The results obtained are like those described in the literature. Examples of flavonoids with EPI activity against expulsion systems present in *S. aureus* are epigallocatechin gallate (EGCg), a compound with a structure similar to (+)-catechin and (−)-epicatechin, Gibbons (2024) biochanin A, 5-methoxyhydnocarpine (Tegos et al., 2011; Stermitz et al., 2000), flavones and flavonoglycans derived from 5-methoxyhydnocarpine (Dashtbani-Roozbehani and Brown, 2021), and baicalein (Dashtbani-Roozbehani and Brown, 2021; Chan et al., 2011).

A despite this, the potential EPI activity observed with the flavonoids studied does not agree with the significant accumulation of BrEt in the *S. aureus* strains obtained with the grape pomace extracts obtained with the solvents, hexane and chloroform. This may be due to the fact that within the grape pomace the phenolic and polyphenolic compounds are the most abundant (Sanhueza et al., 2017; Buican et al., 2025; Abreu et al., 2005). However, non-phenolic compounds such as terpenes and fatty acids may also be present in grapes and their pomace, which can be extracted with non-polar solvents such as *n*-hexane (Buican et al., 2025). Some terpenes have also been described in the literature as able of inhibiting efflux pumps, an example of this is totarol (Smith et al., 2007). Therefore, it is possible that these types of compounds are present in the *n*-hexane and chloroform fractions of the grape pomace extract and that they are the main responsible for the significant inhibitory effect observed in these fractions, considering that when quercetin, the only flavonoid identified in this extract, was tested, it resulted in low inhibitory activity, which does not coincide with that observed when the cells were treated with the extract.

## 4 Conclusion

The grape extracts obtained with *n*-hexane, chloroform and ethyl acetate, in addition to the identified flavonoids (quercetin, (+)-catechin and (−)-epicatechin), showed synergy with ciprofloxacin in all the tested strains. Grape pomace extracts obtained with *n*-hexane and chloroform showed a significant accumulation of EtBr fluorescence, exceeding that of the control inhibitor (CCCP), suggesting potential inhibitory activity against efflux pumps in *S. aureus*. This activity was determined to be nonspecific, meaning it affects more than one efflux pump present in this bacterium. The flavonoids (+)-catechin, (−)-epicatechin and quercetin showed inhibitory activity against efflux pumps in *S. aureus*; however, this activity was lower in the fractions obtained with *n*-hexane and chloroform. The flavonoid (−)-epicatechin showed activity against the NorA efflux pump, widely distributed in *S. aureus* strains. Further molecular expression studies are required to determine whether (−)-epicatechin is able to specifically inhibit NorA efflux pumps.

## Data Availability

The original contributions presented in the study are included in the article/supplementary material, further inquiries can be directed to the corresponding author/s.
